# Wnt3a ligand facilitates autophagy in hippocampal neurons by modulating a novel GSK-3β-AMPK axis

**DOI:** 10.1186/s12964-018-0227-0

**Published:** 2018-04-11

**Authors:** Juvenal A. Ríos, Juan A. Godoy, Nibaldo C. Inestrosa

**Affiliations:** 1 Centro de Envejecimiento y Regeneración UC (CARE UC), Departamento de Biología Celular y Molecular, Facultad de Ciencias Biológicas, Pontificia Universidad Católica de Chile, Alameda 340, PO Box 114-D, Santiago, Chile; 2 Centre for Healthy Brain Ageing, School of Psychiatry, Faculty of Medicine, University of New South Wales, Sydney, Australia; 3 Laboratorio de Fisiología Molecular, Departamento de Ciencias Experimentales y de la Salud, Universidad Pompeu de Fabra, Barcelona, Spain; 4 Centro de Excelencia en Biomedicina de Magallanes (CEBIMA), Universidad de Magallanes, Punta Arenas, Chile

**Keywords:** Neuron, Wnt signaling, AMPK, Autophagy, Metabolism

## Abstract

**Background:**

In the adult central nervous system (CNS), Wnt signaling regulates dendritic structure and synaptic plasticity. The Wnt signaling pathway can be divided into the canonical (β-catenin-dependent) and non-canonical pathways. In the canonical pathway, the binding of canonical ligands such as Wnt3a to the Frizzled receptor induces inactivation of glycogen synthase kinase-3β (GSK-3β), which stabilizes β-catenin and allows its translocation to the nucleus. However, to date, few studies have focused on β-catenin-independent Wnt signaling or explained the underlying mechanisms connecting Wnt signaling to cellular energy metabolism. A recent study demonstrated negative regulation of 5′ adenosine monophosphate-activated protein kinase (AMPK), a major target of GSK-3β that regulates cellular metabolism under diverse conditions. Mainly based on these observations, we evaluated whether Wnt3a ligand modulates autophagy by regulating the GSK-3β/AMPK axis.

**Methods:**

Cultured primary hippocampal neurons and slices of the CA1 region of rat hippocampus were used. GSK-3β inhibition, AMPK activation, PP2Ac expression, and LC3 processing were examined by western blotting. Autophagic compartments were studied using the CYTO-ID® fluorescent probe, and mature autophagosomes were observed via transmission electron microscopy (TEM).

**Results:**

Wnt3a ligand, acting through the Frizzled receptor, promotes the rapid activation of AMPK by inactivating GSK-3β. Biochemical analysis of downstream targets indicated that Wnt3a ligand modulates autophagy in hippocampal neurons.

**Conclusions:**

Our results revealed new aspects of Wnt signaling in neuronal metabolism. First, AMPK is an additional target downstream of the Wnt cascade, suggesting a molecular mechanism for the metabolic effects previously observed for Wnt signaling. Second, this mechanism is independent of β-catenin, suggesting a relevant role for non-genomic activity of the Wnt pathway in cellular metabolism. Finally, these results have new implications regarding the role of Wnt signaling in the modulation of autophagy in neurons, with a possible role in the removal of accumulated intracellular proteins.

## Background

Wnt signaling regulates several biological processes, including brain development and stem cell proliferation, and has been implicated in a whole spectrum of diseases, from cancer to neurodegeneration [1]. Mechanistically, Wnt ligands bind to the Frizzled (Fz) subfamily of transmembrane receptors, and depending on the combination of ligands and receptors, the following pathways can be recognized: the canonical Wnt pathway, which regulates gene transcription through β-catenin accumulation and translocation to the nucleus; and the non-canonical Wnt pathway, which modulates the cytoskeleton via intracellular Ca^2+^ release and activation of small GTPases [2]. In the canonical Wnt pathway, after canonical Wnt ligands bind to the Fz receptor/lipoprotein receptor-related protein 5/6 (LRP5/LRP6) complex, Dishevelled (Dvl) protein is recruited to the membrane, causing the disassembly of the “Wnt destruction complex”, which includes Axin, the tumor suppressor Adenomatous Polyposis Coli gene product (APC), Casein Kinase 1 (CK1), and glycogen synthase kinase-3β (GSK-3β). The inactivation of GSK-3β is the central step of canonical Wnt signaling, which allows β-catenin stabilization and translocation to the nucleus [3]. However, in recent years, new actors have appeared in the canonical Wnt scenario, indicating β-catenin independent pathways that do not require β-catenin accumulation and whose biological effects depend on the rapid modulation of GSK-3β in the cytoplasm. One such pathway is the Wnt/TOR pathway, which derepresses TORC1 activity and promotes protein translation [4]. On the opposite side is the Wnt/STOP branch, which peaks during mitosis and leads to slow protein degradation during cell division [5–7]. In this way, several reports indicate that Wnt promotes the relocalization of ubiquitinated proteins from the proteasome to the endolysosome system via the Wnt/STOP mechanism [8].

To date, data on the role of Wnt signaling at the cellular metabolic level include GSK-3β as a point of intersection with the insulin pathway [9, 10]; transcriptional stimulation of metabolic enzymes such as aldolase, cytidine deaminase, dihydrolipoamide *S*-succinyltransferase, and lysosomal cysteine proteinase [11]; regulation of glycosylation events by the hexosamine biosynthetic pathway [12]; mitochondrial biogenesis in muscle tissue [13]; modulation of mitochondrial dynamics in hippocampal neurons [14, 15]; and more recently, enhanced glucose utilization through glycolysis and the pentose phosphate pathway in cortical neurons [16, 17]. At a systemic level, Wnt signaling plays an extensive role in the regulation of liver, pancreas, and adipose tissue, as well as in metabolic diseases such as type 2 diabetes (T2DM) and metabolic syndrome [18]. Therefore, Wnt signaling is thought to act as a central integrator of metabolic signals from peripheral organs to the brain, representing a novel role for the Wnt signaling pathway in cellular metabolism.

However, the precise mechanisms that connect Wnt signaling with cellular metabolic events remain unclear. In this sense, 5′ adenosine monophosphate-activated protein kinase (AMPK) is a good candidate. This heterotrimeric protein kinase is extensively expressed in all tissue types, including hippocampal neurons [19, 20], acting as a metabolic sensor when the AMP/ATP ratio increases, which leads to AMPK phosphorylation at Thr172. Once activated, AMPK modulates downstream enzymes associated with glycolysis, lipolysis and autophagy; it impacts autophagy by inhibiting the mTORC1 complex [21]. The upstream regulation of AMPK involves two key enzymes, liver kinase B1 (LKB1) and Ca^+ 2^/CaM-dependent protein kinase β (CaMKKβ) [22]. AMPK is returned to its inactive form by dephosphorylation mediated by specific phosphatases, such as the PP2A family (PP2C and PP2A). The PP2A catalytic subunit (PP2Ac) is ubiquitously expressed throughout the brain [23, 24] and is most highly regulated by the proteasomal system, giving it a short half-life [25, 26]. In 2014, Suzuki and colleagues demonstrated a novel negative regulation of AMPK by GSK-3β on Thr479, promoting access to activating phosphorylation (Thr172) by PP2A and subsequent dephosphorylation, resulting in AMPK inactivation. Importantly, these findings were later confirmed by other authors under different conditions [27–30].

Interestingly, several reports have shown that autophagy can negatively modulate the Wnt signaling pathway through Dvl degradation [31–33]. Moreover, we previously hypothesized a link between Wnt signaling and other anti-aging pathways, such as the AMPK-mTOR-autophagy axis, suggesting a possible connection through early events, such as GSK-3β inhibition and/or Ca^+ 2^ release after Wnt activation [34]. In the present work, in hippocampal neurons, Wnt3a ligand modulates AMPK activation through a mechanism dependent on GSK-3β inhibition, and this effect is specific to the Wnt pathway. Additionally, Wnt3a ligand modulates autophagy in the CA1 region of the rat hippocampus.

## Methods

### Culture of rat hippocampal neurons

Rats were housed at the Animal Warehouse Facility of Pontificia Universidad Católica de Chile. The Bioethical and Biosafety Committee of the Faculty of Biological Sciences at our university approved the experimental procedures. Briefly, hippocampi were isolated and dissected from Sprague–Dawley rats at embryonic day 18. Primary rat hippocampal neurons were maintained in Dulbecco’s Modified Eagle’s Medium supplemented with 10% horse serum for 2 h at 37 °C. Then, the medium was substituted with Neurobasal medium supplemented with B27, 100 U/mL streptomycin, and 100 U/mL penicillin and cultured at 37 °C with 5% CO_2_. After 3 and 7 days in vitro (DIV), cells were treated with AraC for 24 h to avoid glial cell growth. Neurons were ready for experimentation at 14 DIV as previously described [14].

### Reagents

Recombinant Wnt3a and Dkk1 were purchased from R&D Systems (McKinley, Minneapolis, USA). Lithium chloride was from Sigma Chemicals (Frankfurt, Darmstadt, Germany). TCS-183 was obtained from Tocris Bioscience (Atlantic Rd., Bristol, UK). Rapamycin was from Enzo Life Technologies (Farmingdale, NY, USA), and metformin hydrochloride was purchased from Abcam Biochemicals® (Cambridge, Cambridgeshire, UK).

### Western blotting and antibodies

Total protein extraction from cultured hippocampal neurons and immunoblot analysis were performed as previously described [35]. The following primary antibodies were used: rabbit anti-pSer9-GSK-3β, anti-GSK-3β, rabbit anti-pThr172-AMPK, mouse anti-AMPKα1 (1:1000), Cell Signaling (Danvers, Massachusetts, USA); mouse anti-AMPKα1/α2 (1:1000), Abcam (Cambridge, Cambridgeshire UK); rabbit anti-PP2Ac (1:1000), Cell Signaling (Danvers, Massachusetts, USA); rabbit anti-LC3B (1:1000), (Danvers, Massachusetts, USA); rabbit anti-β-catenin (1:500), Santa Cruz (Dallas, Texas, USA); and mouse anti-α-actin (1:15000), Sigma (Frankfurt, Darmstadt, Germany). Primary antibodies were recognized using either horseradish peroxidase (HRP)-conjugated goat anti-rabbit antibody (1:7000, Thermo Scientific, Waltham, Massachusetts, USA) or HRP-conjugated rabbit anti-mouse antibody (1:7000, Thermo Scientific, Waltham, Massachusetts, USA). Secondary antibodies were detected via enhanced chemiluminescence using an ECL Plus WB detection system (Little Chalfont, Buckinghamshire, UK). Densitometric analysis was performed using ImageJ software (NIH, USA), and quantification was performed by normalization strategies proposed in 2013 [36].

### Autophagy detection by Cyto-ID® dye staining and immunofluorescence assay

Several previous reports have agreed that the Cyto-ID® Autophagy Detection probe (Farmingdale, NY, USA) labels autophagosomes and have validated this probe as a good estimate of autophagic flux [37, 38]. Dye staining was performed according to the manufacturer’s instructions and as previously described [39, 40]. Hippocampal neurons were grown 14 DIV on coverslips. Cultures were washed and depleted of supplement and antibiotics for 2 h before the experiments were performed. Next, the cultures were exposed to Wnt3a ligand or rapamycin or co-incubated with Wnt3a ligand plus rapamycin for 4 h and stained with the Cyto-ID® probe at 37 °C for 45 min in the dark. Neurons were fixed and immunostained with goat anti-MAP1B (1:250, Santa Cruz, USA) as previously described in our laboratory [35]. Stained cells were photographed on a Zeiss LSM 5 Pascal confocal microscope. Ten micrographs per experimental condition were recorded, and analysis was performed using ImageJ software (NIH, USA).

### Transmission electron microscopy (TEM)

We prepared brain slices from rat brains by cutting transverse slices of 400 μm using a vibrating microtome; slices were maintained under cold artificial cerebrospinal fluid (ACSF), according to a previously described protocol and [41]. The slices were oxygenated in ACSF for more than 1 h at room temperature. For different experimental conditions, the slices were mounted and treated in 6-well plates with constant oxygenation. After treatment, the slices were transferred to an Eppendorf tube and fixed for TEM [42, 43]. Ultra-thin sections were examined using a Phillips Tecnai 12 transmission electron microscope at 80 kV at the Electron Microscope Facility of the Faculty of Biological Sciences, Pontificia Universidad Católica de Chile, Santiago, Chile. For morphological analysis of autophagosomal structure, we captured 25 digital images per condition at 16.500–20.500× magnification and counted autophagic vesicles (AVs) using the ultrastructural parameters according to the procedures described by the Nixon laboratory [44–46].

### Statistical analysis

Data are presented as the mean ± SEM. The number of experimental replicates is indicated by “n”. ANOVA followed by uncorrected Fisher’s LSD post hoc test was used to assess statistically significant differences. Statistical analysis was performed using GraphPad Prism 6.0v (GraphPad Software, Inc., La Jolla, CA).

## Results

### Wnt3a ligand modulates AMPK phosphorylation levels in hippocampal neurons

**Fig. 1:**
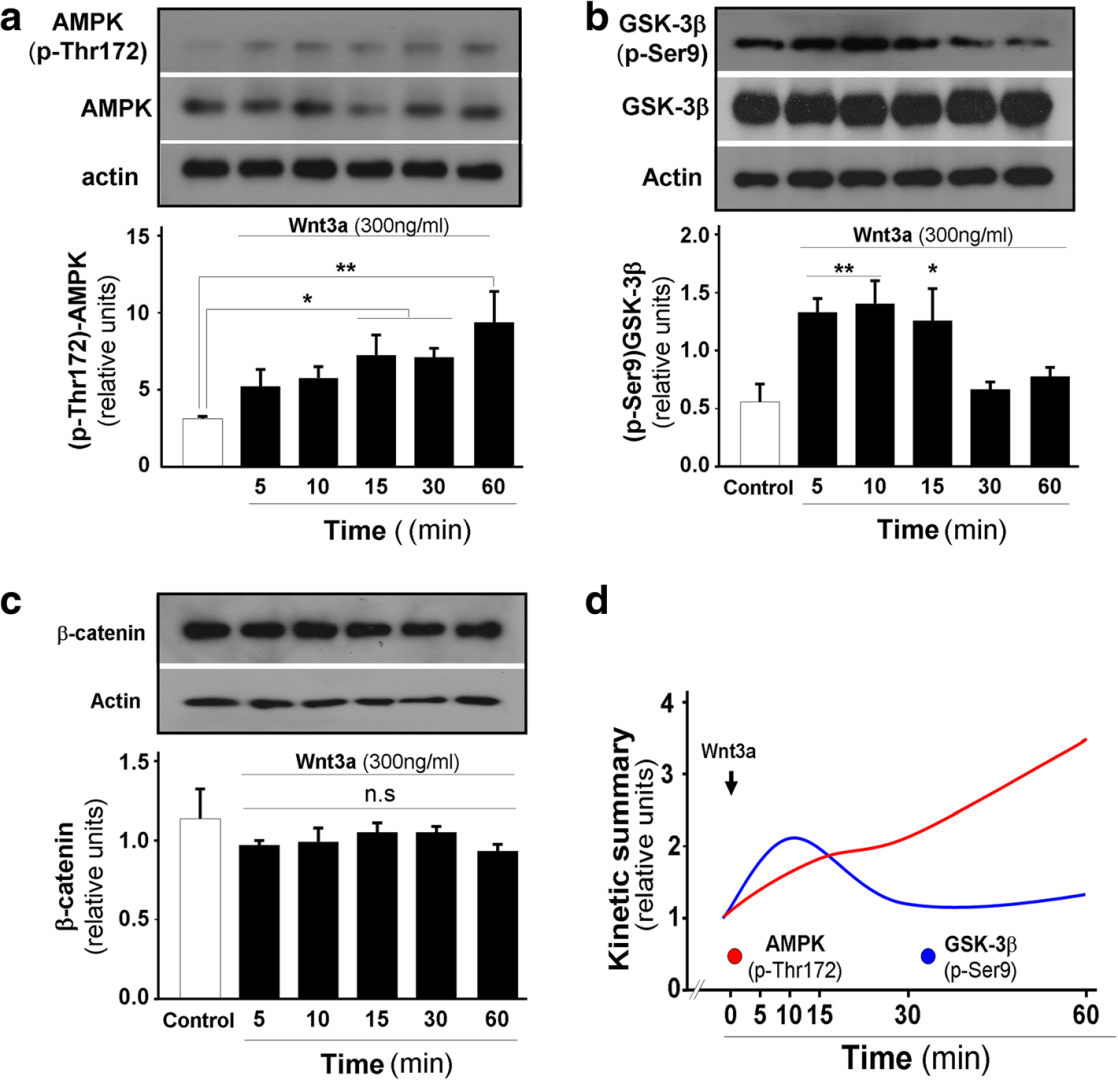
Wnt3a ligand modulates AMPK activity in hippocampal neurons. **a** Wnt3a ligand significantly increases AMPK phosphorylation at Thr172 between 15 and 60 min of treatment. **b** GSK-3β shows significant inhibition (phosphorylation at Ser9) from 5 to 15 min of treatment with Wnt3a ligand. **c** β-catenin did not accumulate in neurons over the same time course. **d** Graphic showing kinetic summary of AMPK activation and GSK-3β inhibition. The data are expressed as the mean ± S.E.M. of *n* = 3 independent experiments, *p < 0.05, **p < 0,001, n.s, non-significant

We assessed whether stimulation with the canonical Wnt3a ligand modulates AMPK activity in hippocampal neurons. We treated neurons with 300 ng/ml of Wnt3a ligand and monitored AMPK activity by assessing Thr172 phosphorylation over 60 min. AMPK phosphorylation significantly increased (2–3-fold) from 15 to 60 min (Fig. 1a). In parallel, we evaluated GSK-3β inactivation by measuring Ser9 phosphorylation. GSK-3β inactivation occurred very early, from 5 to 15 min (Fig. 1b). Interestingly, the levels of β-catenin protein, the main protein that accumulates after inhibition of GSK, were unchanged (Fig. 1c). We summarize these experiments, the time course of AMPK activation and GSK-3β inhibition in a simplified graphic. Following treatment with the canonical Wnt3a ligand, GSK-3β activity began to decrease at 15 min, with a concomitant increase in AMPK activity, which was maintained during our experimental conditions, without accumulation of β-catenin (Fig. 1d). These results suggest that the inactivation of GSK-3β allows the activation of AMPK under our experimental conditions.

### Wnt3a ligand-triggered AMPK activation requires canonical FzR-LRP5/6-mediated GSK-3β inhibition and does not affect the turnover of phosphatase PP2Ac

**Fig. 2:**
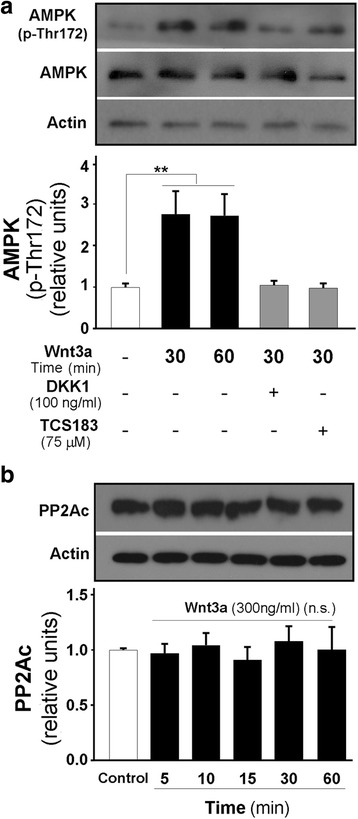
Increased AMPK activity by Wnt3a ligand requires canonical FzR-LRP5/6 and GSK-3β inhibition and does not affect the turnover of phosphatase PP2Ac. **a** After 30 min, Wnt3a ligand induces AMPK activation, which was abolished by Dkk1 and TCS183 pretreatment. **b** Measurement of PP2A catalytic subunit levels indicates no changes during time-course treatment. The data are expressed as the mean ± S.E.M. of three independent experiments, **p < 0.001, n.s., non-significant

The canonical Wnt pathway signals through the Fz receptor along with LRP5/6. This signaling can be antagonized using Dickkopf 1 (Dkk1), an LRP5/6 antagonist, which prevents GSK-3β inactivation and leads to β-catenin destruction. In our experiments, AMPK phosphorylation significantly increased following the addition of 300 ng/ml Wnt3a ligand at 30 and 60 min of treatment. Interestingly, decreased AMPK activity was observed in the presence of the canonical Wnt inhibitor Dkk1 (Fig. 2a). Similarly, decreased AMPK phosphorylation was detected when we used synthetic peptide TCS-183, which blocks GSK-3β autoinhibition (Fig. 2a). To evaluate how Wnt3a ligand was acting through the known mechanism of AMPK regulation, we measured the specific phosphatase PP2Ac, which dephosphorylates and inhibits AMPK in the presence of Wnt3a ligand. No significant changes were observed in the levels of the PP2A catalytic subunit in the presence of 300 ng/ml Wnt3a ligand in our experiments (Fig. 2b).

### Inhibiting GSK-3β with lithium induces AMPK activity without altering PP2Ac levels in hippocampal neurons

**Fig. 3:**
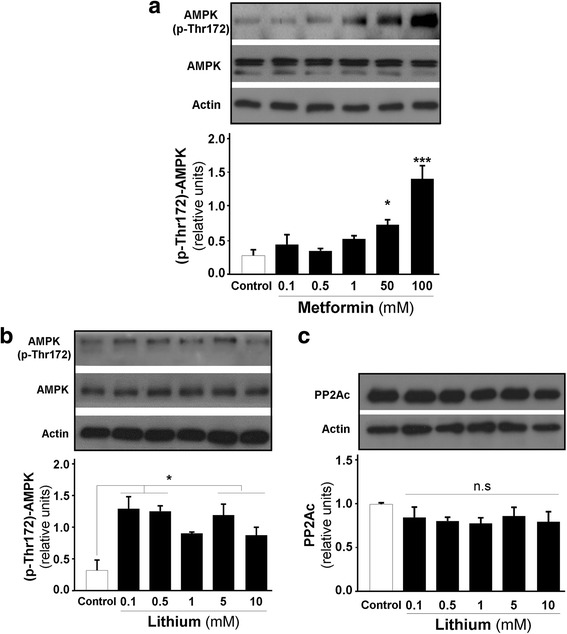
Pharmacological inhibition of GSK-3β by lithium induces AMPK activity without altering PP2Ac levels. **a** Neurons were treated with metformin for 1 h at 37 °C for dose-response experiments. **b** Hippocampal neurons were treated with lithium (from 0.1 to 10 mM) for 1 h at 37 °C. AMPK was activated at low doses of lithium. **c** Measurement of the levels of the catalytic subunit of PP2A under lithium treatment; no changes were found after lithium treatment. The data are expressed as the mean ± S.E.M. of *n* = 3 independent experiments, *p < 0.05, ***p < 0.0001. n.s., non-significant

We used lithium, a pharmacological inhibitor of GSK-3β, that produces the stabilization of β-catenin to verify GSK-3β-dependent activation of AMPK [47, 48]. In parallel experiments, metformin, a conventional drug for indirectly activating AMPK, was used [49]. Metformin strongly activated AMPK in hippocampal neurons; AMPK showed a significant increase between 50 and 100 mM of metformin in cultured hippocampal neurons (Fig. 3a). With lithium, AMPK activity increased in a dose-dependent manner after 1 h at 37 °C (Fig. 3b). Additionally, we studied the effects of lithium on PP2Ac levels, which did not change after lithium treatment under the same conditions (Fig. 3c).

### Wnt3a ligand modulates autophagy in hippocampal neurons

**Fig. 4:**
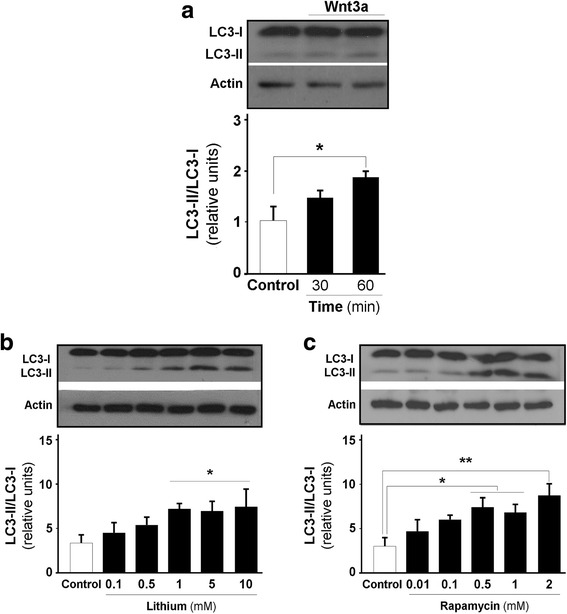
Wnt3a ligand enhances autophagy initiation in hippocampal neurons. **a** Wnt3a ligand enhanced autophagy initiation, promoting maturation from LC3I to LC3II at 37 °C for 60 min in hippocampal neurons. In parallel cultures, (**b**) dose-response curves for lithium (60 min) and (**c**) rapamycin (120 min) were used as controls, and both showed increased processing of LC3 in hippocampal neurons. The graphs show the ratio between the LC3II/LC3I fraction that was normalized to actin. The data are expressed as the mean ± S.E.M. of *n* = 3 independent experiments, *p < 0.05, **p < 0.001

Downstream of AMPK activation, autophagy is typically triggered upon mTORC1 inhibition [50, 51]. Autophagy can be divided into three main molecular steps, initiation, elongation and termination, which occur at different times depending on the cell type [52]. Using three different experimental approaches, we evaluated whether Wnt3a ligand regulates any of the autophagic steps in hippocampal neurons at different time points. For the initiation step, we evaluated the processing of LC3 by western blotting in hippocampal neurons treated with Wnt3a ligand for 30–60 min. Compared with the control, we observed a 50% increase in LC3II after Wnt3a ligand after 30 min and resulted in a 75% increase after 60 min of treatment (Fig. 4a). Additionally, experiments with lithium enhanced this initiation step in hippocampal neurons after 60 min with increasing doses of lithium (Fig. 4b). In parallel, we used rapamycin, a known inducer of autophagy initiation, in a dose-response curve and verified the expected increase in LCR3 processing. We observed the expected enhancement of autophagy initiation after 60 min of rapamycin treatment (Fig. 4c).

**Fig. 5:**
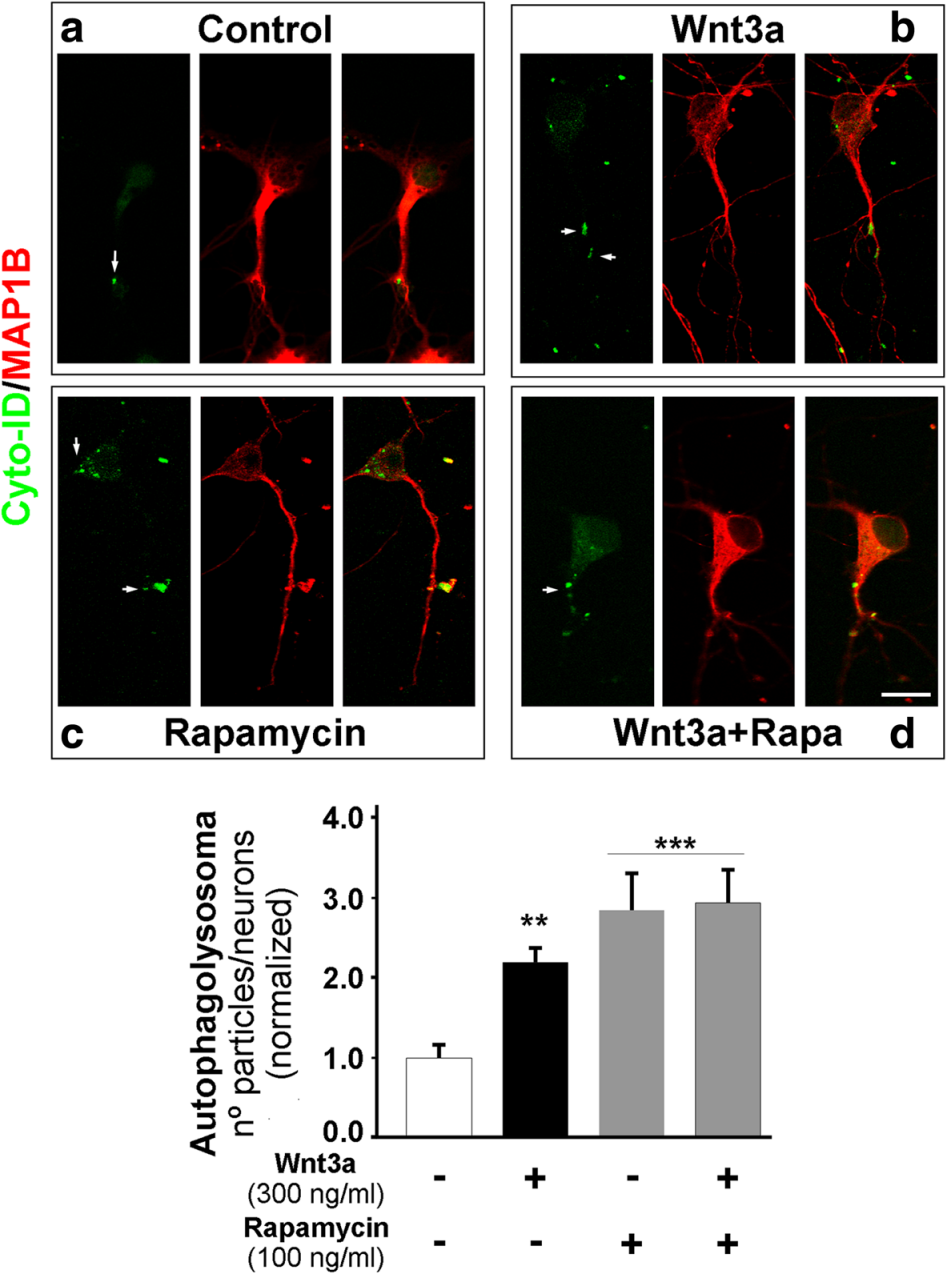
Wnt3a ligand increases autophagic puncta in hippocampal neurons. The micrographs show representative neurons stained with the Cyto-ID probe and MAP1B to visualize autophagosomal compartments after 4 h of culture at 37 °C under the following culture conditions: control neurons (**a**); Wnt3a ligand, (**b**); rapamycin, (**c**); and Wnt3a ligand plus rapamycin (**d**). Green puncta on neurons were quantified (see white arrow). Compared with the control, treatment with Wnt3a ligand produced a 2-fold increase, while both rapamycin alone and co-treatment with Wnt3a ligand plus rapamycin produced a similar 3-fold increase over the control. The data are expressed as the mean ± S.E.M. of three independent experiments, (20–25 neurons for each condition and normalized with the control). **p < 0.001; ***p < 0.0001. The scale bar represents 10 nm

To study the elongation step in cultured hippocampal neurons, we used Cyto-ID (autophagy detection kit), which highlights autophagosomal compartments with green fluorescence [39]. Compared with the control, Wnt3a ligand induced a significant increase (2-fold) in the number of autophagolysosomes**/**neuron (Fig. 5, white arrow). Rapamycin was used as a positive control, and a 3-fold increase over the control was observed. Interestingly, Wnt3a ligand plus rapamycin co-treatment did not show a synergistic effect (Fig. 5, see graph, fourth gray bar).

**Fig. 6:**
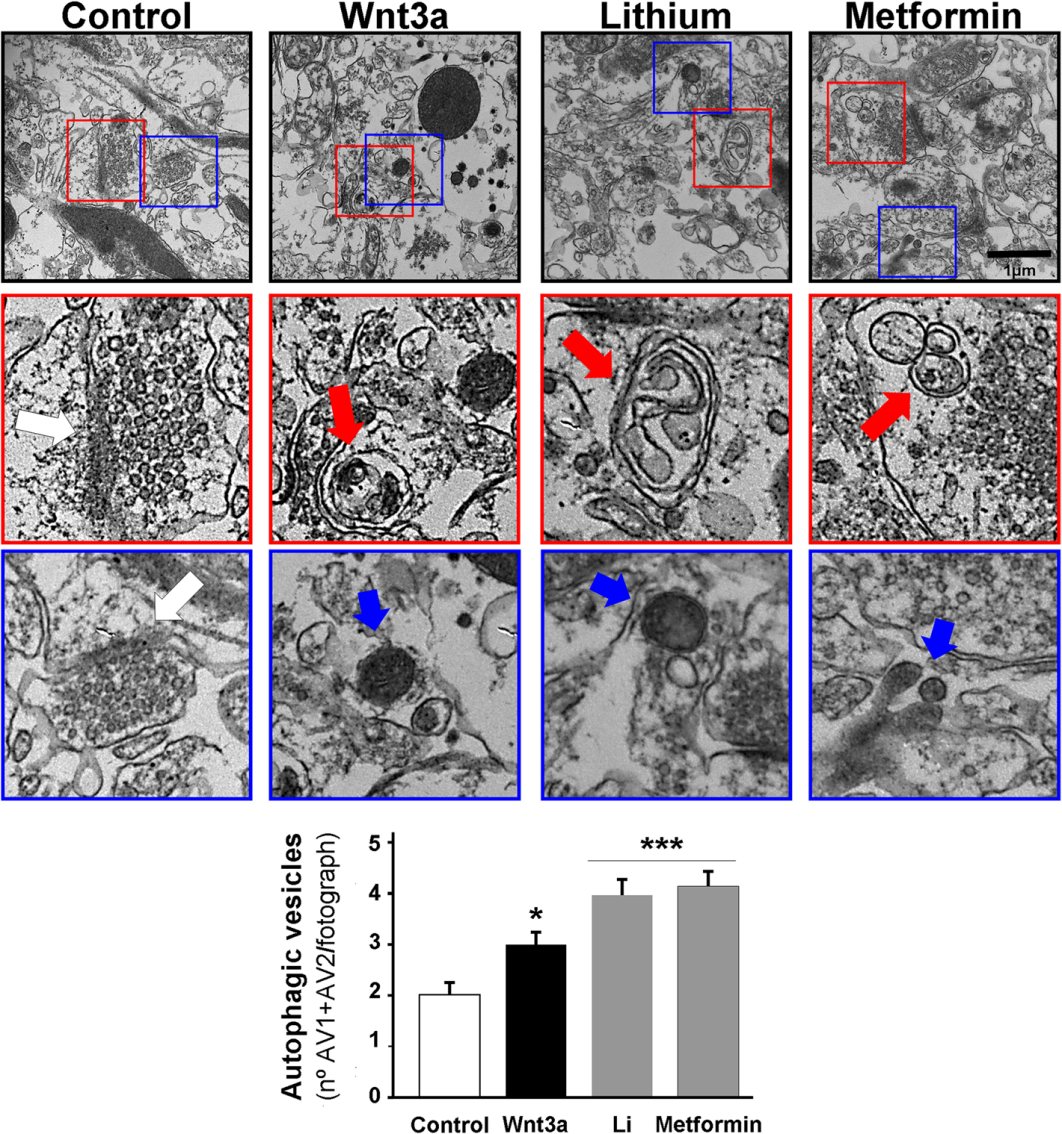
Wnt3a ligand promotes autophagic vesicle formation in the CA1 region of the rat hippocampus. Hippocampal slices were oxygenated in ACSF and treated for 4 h with Wnt3a ligand, lithium and metformin. Ultrastructural analyses were performed to visualize autophagic vesicles (AVs) (see graph). In the control slices, normal synapses containing synaptic vesicles are shown with the postsynaptic density (first column; see white arrow). The number of vesicles per image was determined by counting two vesicle types. Early vesicles (AV1) were defined by a large double membrane containing amorphous material (Wnt3a ligand treatment; second column, zoomed-in red square), and late autophagic vesicles (AV2) were characterized by a single-membrane vesicle containing electron-dense intraluminal material (e.g., zoomed-in Wnt3a ligand treatment; blue square). The representative photograph at 20500× magnification shows regions of interest. The data are expressed as the mean ± S.E.M. of 20–25 micrographs of three independent experiments, *p < 0.05, ***p < 0.0001; the scale bar represents 1 μm

Finally, we used TEM to determine the presence and number of autophagolysosomal structures in the rat hippocampal CA1 region, which is rich in synapses (control slices, see white arrow) where Wnt ligands have an effect on learning/memory processes. Wnt3a ligand has an especially marked influence on fear memory acquisition and consolidation [53]. The number of AVs was determined according to morphological parameters. Early vesicles were defined by a large double-membrane with amorphous material inside (see red arrow), and mature vesicles were defined by a simple membrane with electron-dense intraluminal material (see blue arrow) [45]. Compared with slices treated with the control, slices treated with Wnt3a ligand for 4 h showed up to a 50% increase in the number of AVs. A similar effect was observed for lithium, which was used as a positive control for the activation of the canonical Wnt signaling pathway, and metformin, which activates AMPK. In our experiments, compared with the control, both lithium and metformin showed 2-fold increases in the number of AVs (Fig. 6).

## Discussion

To date, Wnt signaling has been shown to play an extensive role in neuroprotection that is particularly concentrated in the synaptic region [54–56]. Wnt3a ligand and lithium pretreatment, both recognized as canonical Wnt activators, have also been demonstrated to protect neurons from general Aβ-induced neurotoxic damage [57]. More recently, studies in our laboratory have shown that Wnt3a ligand protects mitochondrial structure and function from Aβ challenge [43, 58]. However, these beneficial effects of Wnt ligands occur hours after pretreatment and could therefore be through β-catenin-mediated gene transcription. Interestingly, Wnt3a ligand treatment could prevent Aβ-induced tau phosphorylation in hippocampal neurons through GSK-3β-mediated inhibition [59]. Similarly, Dkk1 knockdown prevented the hyperphosphorylation of tau in cortical neurons exposed to Aβ [60, 61]. These latter findings have highlighted the remarkable role of GSK-3β as a potential master regulator of the rapid β-catenin-independent effects of the Wnt signaling pathway.

Indeed, previous reports have described an alternative branch of Wnt/GSK-3β signaling, including the prevention of aberrant tau and microtubule-associated protein 1B (MAP1B) phosphorylation mediated by Wnt3a ligand and Wnt7a treatment, respectively [59, 62]. Moreover, in this sense, metabolic studies indicated that Wnt3a ligand stimulates glucose uptake and the glycolytic rate in cortical neurons without β-catenin accumulation in the cytoplasm [16].

**Fig. 7:**
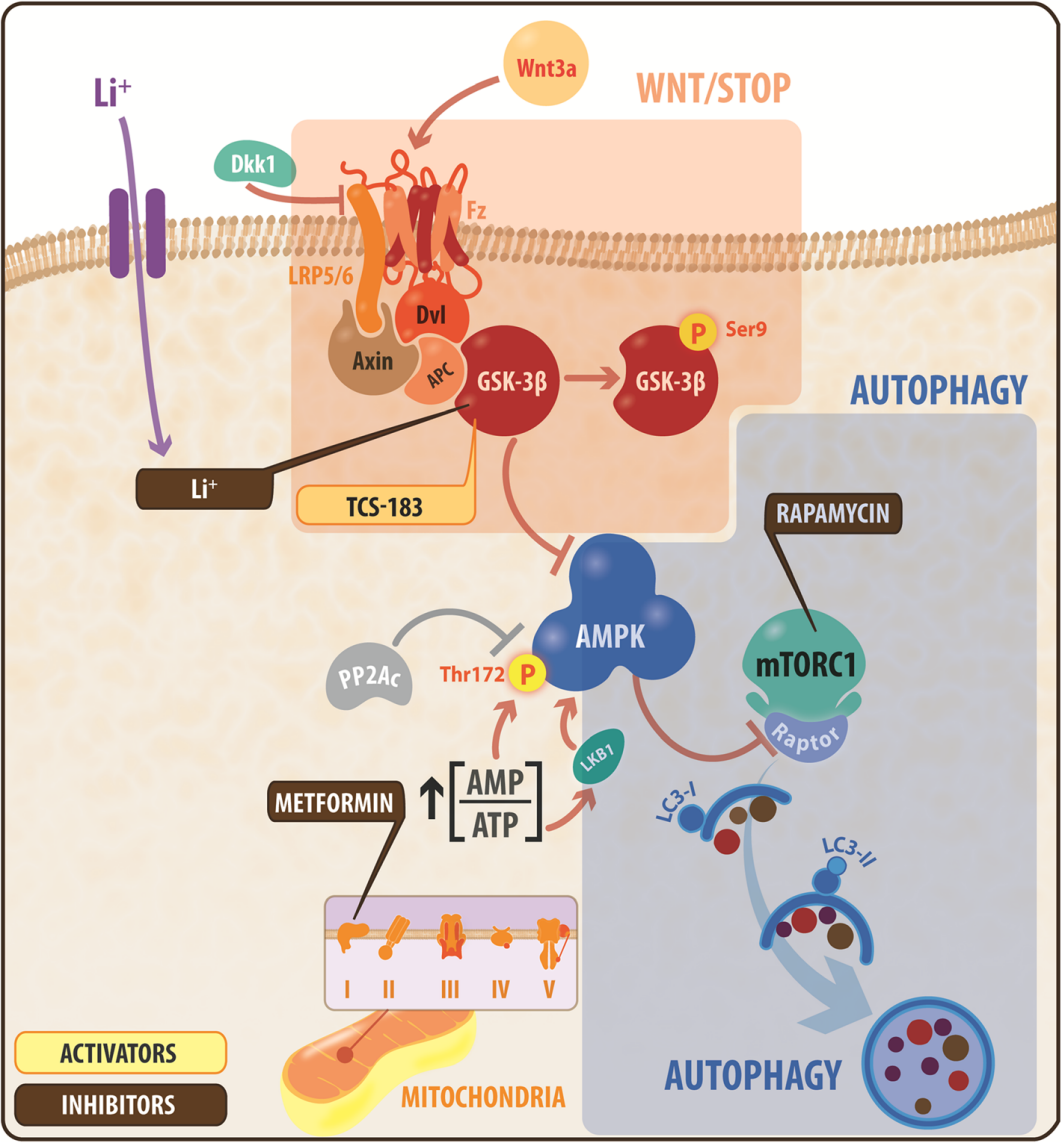
Wnt3a ligand regulates autophagy via the Wnt/STOP mechanism, modulating the GSK-AMPK axis in hippocampal neurons. In this scheme, we summarize our findings in conjunction with previous knowledge. Wnt3a ligand binding to the Frizzled receptor requires the presence of the LRP5/6 canonical co-receptor, leading to GSK-3β inhibition (Ser9 phosphorylation). This effect also occurs in the presence of lithium or TCS-183, which are both known inhibitors of GSK-3β. AMPK, a key regulator of cellular metabolism, was inhibited by basal GSK-3β activity; therefore, GSK-3β inhibition promotes AMPK activation. In addition, PP2Ac, the major AMPK phosphatase in the brain, did not affect this regulation mediated by the Wnt pathway (shown in gray). Lithium, rapamycin and metformin are indirect activators of AMPK through the inhibition of mitochondrial complex I, which leads to decreased ATP levels and results in an increased [AMP/ATP] ratio. The increased AMP/ATP ratio allows access to the two main upstream AMPK kinases, LKB1 and CaMKKβ. Downstream, we show that Wnt3a ligand promotes the initiation of autophagy as illustrated by LC3 maturation from LC3I to LC3II, as well as increased accumulation of autophagosome/autophagolysosome structures in cultured hippocampal neurons and the CA1 region of rat hippocampus

In the present work, for the first time, we demonstrate that Wnt3a ligand can modulate AMPK activation and autophagy in hippocampal neurons; these two pathways are directly connected (Fig. 7). According to our experiments, this effect is specific to the canonical Wnt ligand, which was demonstrated by the use of Dkk1 or TCS-183 inhibitors. The use of these inhibitors abolished AMPK activity. Moreover, the use of these Wnt signaling inhibitors strongly suggests a GSK-3β-dependent mechanism for the observed AMPK phosphorylation (Fig. 2a). Importantly, our findings suggest fast and non-genomic effects of canonical Wnt signaling activation, which can be defined as fast, β-catenin-independent molecular events in hippocampal neurons [4, 63].

However, other regulatory mechanisms for the Wnt3a/AMPK axis have been described, e.g., a rapid influx of Ca^+ 2^ from the extracellular space after Wnt3a ligand treatment potentially suggests an alternative pathway for AMPK activation through the Ca^+ 2^-Calmodulin-CaMKKβ axis [64–66].

Our findings also offer new insights regarding Wnt-autophagy crosstalk because Wnt3a ligand could modulate autophagy in cultured hippocampal neurons. According to assays using the Cyto-ID probe, Wnt3a ligand significantly increased autophagolysosomal staining, but Wnt3a ligand plus rapamycin behaved similarly to rapamycin alone and did not show any synergistic activity or inhibition of the Wnt pathway activated by a canonical Wnt ligand (Fig. 5). Thus, our findings suggest the possible involvement of the Wnt/STOP branching cascade, according an algorithm for dissecting Wnt pathways recently proposed by Acebron and Niehrs in 2016 [4].

Nevertheless, autophagy may be regulated by mTOR-dependent and mTOR-independent pathways that respond to different metabolic conditions [67]. In the case of the mTOR-independent pathway, the activation could be mediated by inositol, calcium signaling or beclin1 pathways, among others [67]. Autophagy exerts control over the activation of the Wnt signaling by degrading ubiquitylated Dvl2 aggregates through the autophagy-lysosome pathway [31–33].

Although the Wnt pathway activates mTORC1 through GSK-3β following non-canonical Wnt/STOP signaling [68]. By the other way, Wnt/β-catenin pathway acting as a negative regulator of both basal and stress-induced autophagy in cancer cells [69], both phenomena occur in mitotic cells. Our neuronal model shows substantial differences because hippocampal neurons are quiescent cells with different metabolic requirements. In this regard, the Wnt pathway, as well as other molecular pathways such as Forkhead box O (FOXO) transcription factors, trigger different and coordinate responses to environmental changes, growth factor deprivation, metabolic stress (starvation) and oxidative stress; the results of this signaling depend on the physiological/pathological state of the cell [70].

Our studies allow us to suggest a hypothetical feedback loop between the Wnt signaling pathway and autophagy that positively modulates autophagy.

Although further research is needed, we believe that our results suggest a novel GSK-3β/AMPK fast route in which Wnt signaling activated by canonical ligands has effects that do not involve the stabilization of β-catenin. Moreover, non-genomic activities, particularly the modulation of autophagy, may be a relevant player in the clearance of toxic intracellular protein accumulation, such as neurofibrillary tangles or intracellular Aβ species, which are highly relevant in different neuropathological conditions, particularly in Alzheimer’s disease.

## Conclusions

In summary, our results highlight at least three novel aspects of the influence of the Wnt pathway on hippocampal neuronal metabolism. First, our results reveal AMPK as an additional actor downstream of the Wnt signaling cascade, suggesting a molecular explanation for the underlying metabolic effects previously observed after Wnt ligand treatment. Second, this mechanism is probably independent of β-catenin, suggesting a role for the non-genomic activity of the Wnt pathway. Finally, our data suggest a novel role for the Wnt pathway in modulating the process of autophagy, which is a pivotal mechanism for preventing the accumulation of toxic intracellular proteins.
